# The “Leafing Intensity Premium” Hypothesis and the Scaling Relationships of the Functional Traits of Bamboo Species

**DOI:** 10.3390/plants13162340

**Published:** 2024-08-22

**Authors:** Weihao Yao, Peijian Shi, Jinfeng Wang, Youying Mu, Jiajie Cao, Karl J. Niklas

**Affiliations:** 1 Co-Innovation Center for Sustainable Forestry in Southern China, Bamboo Research Institute, Nanjing Forestry University, #159 Longpan Road, Nanjing 210037, China; whyao@njfu.edu.cn (W.Y.); jfwang@njfu.edu.cn (J.W.); muyouying@njfu.edu.cn (Y.M.); 2 College of Landscape Architecture, Nanjing Forestry University, #159 Longpan Road, Nanjing 210037, China; 3 School of Integrative Plant Science, Cornell University, 236 Tower Road, Ithaca, NY 14853, USA

**Keywords:** diminishing returns, light-capture, leaf fresh mass, leaf size, scaling theory

## Abstract

The “leafing intensity premium” hypothesis proposes that leaf size results from natural selection acting on different leafing intensities, i.e., the number of leaves per unit shoot volume or mass. The scaling relationships among various above-ground functional traits in the context of this hypothesis are important for understanding plant growth and ecology. Yet, they have not been sufficiently studied. In this study, we selected four bamboo species of the genus *Indocalamus* Nakai and measured the total leaf fresh mass per culm, total non-leaf above-ground fresh mass, total number of leaves per culm, and above-ground culm height of 90 culms from each species. These data were used to calculate leafing intensity (i.e., the total number of leaves per culm divided by the total non-leaf above-ground fresh mass) and mean leaf fresh mass per culm (i.e., the total leaf fresh mass per culm divided by the total number of leaves per culm). Reduced major axis regression protocols were then used to determine the scaling relationships among the various above-ground functional traits and leafing intensity. Among the four species, three exhibited an isometric (one-to-one) relationship between the total leaf fresh mass per culm and the total non-leaf above-ground fresh mass, whereas one species (*Indocalamus pumilus*) exhibited an allometric (not one-to-one) relationship. A negative isometric relationship was found between the mean leaf fresh mass per culm and the leafing intensity for one species (*Indocalamus pedalis*), whereas three negative allometric relationships between mean leaf fresh mass per culm and leafing intensity were observed for the other three species and the pooled data. An exploration of the alternative definitions of “leafing intensity” showed that the total number of leaves per culm divided by the above-ground culm height is superior because it facilitates the non-destructive calculation of leafing intensity for *Indocalamus* species. These results not only confirm the leafing intensity premium hypothesis for bamboo species but also highlight the interconnected scaling relationships among different functional traits, thereby contributing to our understanding of the ecological and evolutionary significance of leaf size variation and biomass investment strategies.

## 1. Introduction

Leaves, as the primary photosynthetic organs in most vascular plants, play an indispensable role in plant growth and development [1,2], and the size of leaves has a marked effect on various biological processes, such as reproduction, survival, and ecosystem function [3,4,5]. Thus, the natural variation in leaf size and its ecological and evolutionary significance have long attracted the attention of researchers in a variety of disciplines [6].

Yet, the surface area of the leaf lamina varies over six orders of magnitude across terrestrial plants [6,7]. Previous explanations of this inter-specific leaf size variation include adaptations to herbivory [8,9] and physiological optimization strategies under different environmental conditions affecting photosynthesis, gas exchange, energy flux, and/or water use efficiency [10,11,12,13]. For example, species surviving in shaded or well-watered environments typically have large laminae that can maximize light interception and carbon acquisition capabilities, while simultaneously reducing the negative effects of shading by the upper canopy [10]. Importantly, an ancillary non-mutually exclusive hypothesis for the variation in leaf size posits a trade-off between leaf size and leaf number per unit shoot size referred to as the “leafing intensity premium” hypothesis [14,15,16,17].

This premise proposes that leaf size variation results from selection acting on different leafing intensities (and thus self-shading), defined as the number of leaves per unit shoot (or stem) volume or mass. According to this hypothesis, species with high leafing intensity (and thus high self-shading) tend to have comparatively small leaves, whereas species with low leafing intensity (and thus low self-shading) tend to have large leaves. Previous studies have demonstrated that leaf size and leafing intensity exhibit a negative scaling relationship across various habitats [15,18], forest successional stages [19], and diverse canopy light environments [20,21]. Additionally, the trade-off between leaf size and mass-based leafing intensity depends on the biomass investment (leaves vs. shoots or stems) [20]. Biomass investment is considered a critical functional trait because it reflects the ability of an organism to use and optimize the allocation of material resources [22] to cope with different environments [23].

Scaling theory, arising from biomass partitioning theory, has identified a number of trade-offs in the allocation of resources among various physiological and ecological functional traits and has provided considerable insights into biomass allocation patterns [24,25]. In the context of the leafing intensity premium hypothesis, it has also shed light on numerous functional traits, such as lamina mass vs. petiole mass, leaf mass vs. lamina area, perianth mass vs. perianth area, and tree height vs. diameter at breast height [26,27,28,29,30]. For example, the scaling exponent for the leaf mass vs. leaf area scaling relationship typically exceeds unity, indicating that leaf area fails to increase proportionally with increasing leaf mass, a phenomenon called “diminishing returns” [7,27].

Here, we use scaling theory to explore the leafing intensity premium hypothesis of an important monocot genus, *Indocalamus* Nakai, a common bamboo growing in the rural areas of southern China, which is known for their cold resistance [31]. *Indocalamus* was also selected for the study because it is of significant ecological value, its broad ecological range, and the fact that species within the genus manifest considerable differences in the number of leaves per culm. For example, *I. longiauritus* often dominates forest understories, providing habitats for numerous species of birds, lizards, and insects [32]. Despite its ecological significance and morphological diversity, there are currently limited studies on the scaling relationship among the above-ground functional traits of *Indocalamus* species and their leafing intensity. To bridge this gap, we selected four *Indocalamus* species and measured the total leaf fresh mass per culm (TLM), total non-leaf above-ground fresh mass (TNLM), above-ground culm height (*H*), and the total number of leaves per culm (*N*). These data were then used to determine the scaling relationships between TLM and TNLM, as well as the scaling relationship between mean leaf fresh mass per culm (MLM), defined as TLM/*N*, and leafing intensity, defined as *N*/TNLM.

An important consideration in this study was how to define “leafing intensity” because alternative definitions have been advanced. Traditionally and most often, leafing intensity is defined as the total number of leaves per shoot divided by total non-leaf above-ground volume or mass of the shoot [14,20,33]. Given that shoot volume and mass are usually positively correlated with plant height (*H*), we tested whether leafing intensity could also be defined as the total number of leaves per shoot divided by above-ground shoot height. If this alternative definition can effectively quantify leafing intensity, it would facilitate the non-destructive calculation of leafing intensity for many plants. To this end, we compared the performance of leafing intensity quantified as *N*/*H* with the traditional metric (i.e., *N*/TNLM).

## 2. Materials and Methods

### 2.1. Sampling Site and Data Acquisition

In early July 2014, along the Verdant Bamboo Road at Nanjing Forestry University (32.08 °N, 118.82 °E), we collected four species of the genus *Indocalamus* Nakai: *Indocalamus barbatus* McClure, *Indocalamus pedalis* (Keng) P. C. Keng, *Indocalamus pumilus* Q. H. Dai and C. F. Keng, and *Indocalamus victorialis* P. C. Keng. Ninety culms were sampled for each species. The collection of specimens took advantage of the ubiquity of the environmental conditions in Nanjing Forestry University and the general region of Nanjing, which belongs to the subtropical region. Based on climate data from 2014 (Source: China Meteorological Administration [Available online: https://www.cma.gov.cn/en/ (accessed on 12 August 2024)]), the mean inter-annual precipitation was 1091.1 mm, the mean annual temperature was 16.4 °C, the mean annual humidity was 74%, and the mean annual sunshine duration was 1863.8 h. The soil type is predominantly mountain yellow-brown and grey-brown soils, which are acidic to slightly acidic [34]. All plants were sampled from the same location. We measured above-ground culm height (*H*), total leaf fresh mass per culm (TLM), and total non-leaf fresh mass per culm (TNLM) using an electronic scale with a precision of 0.01 g (JM-A3002; Chaozeheng Equipment Company Limited, Zhuji, Zhejiang, China). The total number of leaves per culm (*N*) was also recorded, from which the leafing intensity (i.e., *N*/TNLM) and mean leaf fresh mass (i.e., MLM = TLM/*N*) per culm were calculated. The morphological and agronomic characteristics of the four species are shown in Table 1.

### 2.2. Data Analysis

The data for any two interdependent biological measures were analyzed using power law functions taking the form of

(1) $$Y_{1}=\beta Y_{2}^{\alpha }$$

where *Y*_1_ and *Y*_2_ are any two interdependent variables (e.g., plant height and mass), β is the normalization constant, and α is the scaling exponent of the *Y*_1_ vs. *Y*_2_ relationship [26]. To stabilize variance, that raw data were log–log transformed to yield power law functions taking the form of

(2) y=γ+αx

where *y* = ln (*Y*_1_), *x* = ln (*Y*_2_), and γ = ln (β). The parameters γ and α were determined using reduced major axis regression protocols [26,35]. The bootstrap percentile method [36,37], employing 3000 bootstrapping replicates, was used to test the significance of the difference between any two estimated scaling exponents of *Y*_1_ vs. *Y*_2_. The difference between two sets of bootstrap replicates of slopes was determined using the 95% confidence intervals (CIs) of the replicates. If the 95% CIs do not include 0, a significant difference exists; otherwise, there is no significant difference between the two scaling exponents [36,37]. All calculations were performed and figures constructed using software R (version 4.2.0) [38].

## 3. Results

**Figure 1:**
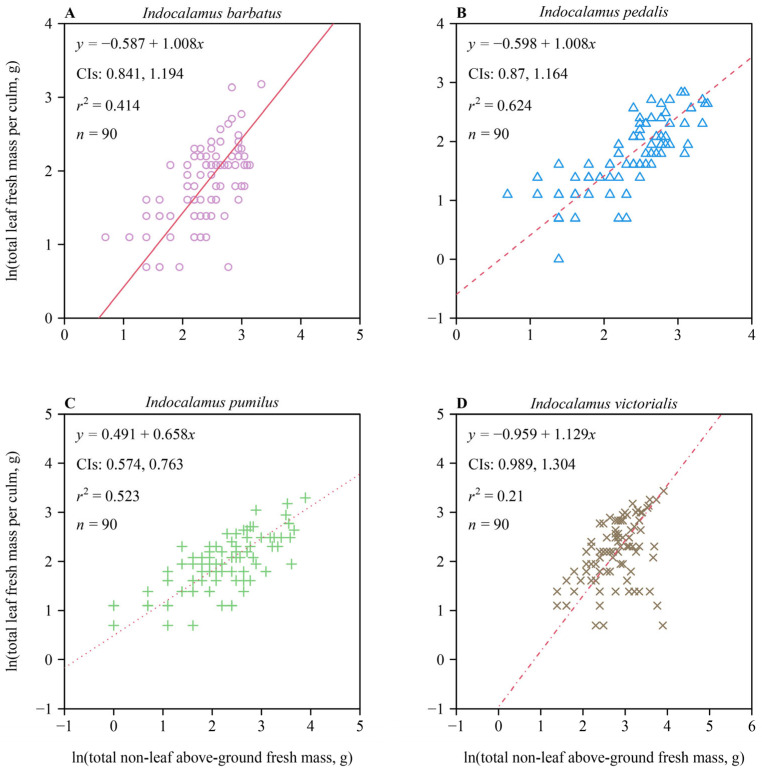
Fitted results for bivariate plots of total leaf fresh mass per culm vs. non-leaf above-ground fresh mass per culm on a log–log scale for four species of *Indocalamus*: *I. barbatus* (**A**), *I. pedalis* (**B**), *I. pumilus* (**C**), and *I. victorialis* (**D**). The red lines are regression curves; CIs denote the 95% confidence intervals of the slope; *r*^2^ is the coefficient of determination; and *n* is the number of culms sampled for each species.

A statistically significant bivariate scaling relationship between TLM and TNLM was observed for each of the four *Indocalamus* species (Figure 1). The 95% CIs of the scaling exponents of TLM vs. TNLM, obtained using the bootstrap percentile method, included unity for three out of the four species (Figure 1A,B,D), indicating that increases in TNLM keep pace with increases in TLM for the three species (i.e., *I. pedalis*, *I. pumilus*, and *I. victorialis* manifested one-to-one scaling relationships). The exception, *I. pumilus*, had an upper bound of the 95% CIs of the TLM vs. TNLM scaling exponent that was smaller than unity (Figure 1C), indicating that increases in TLM did not keep pace with the increase in TNLM, i.e., an allometric scaling relationship was observed for *I. pumilus*.

**Figure 2:**
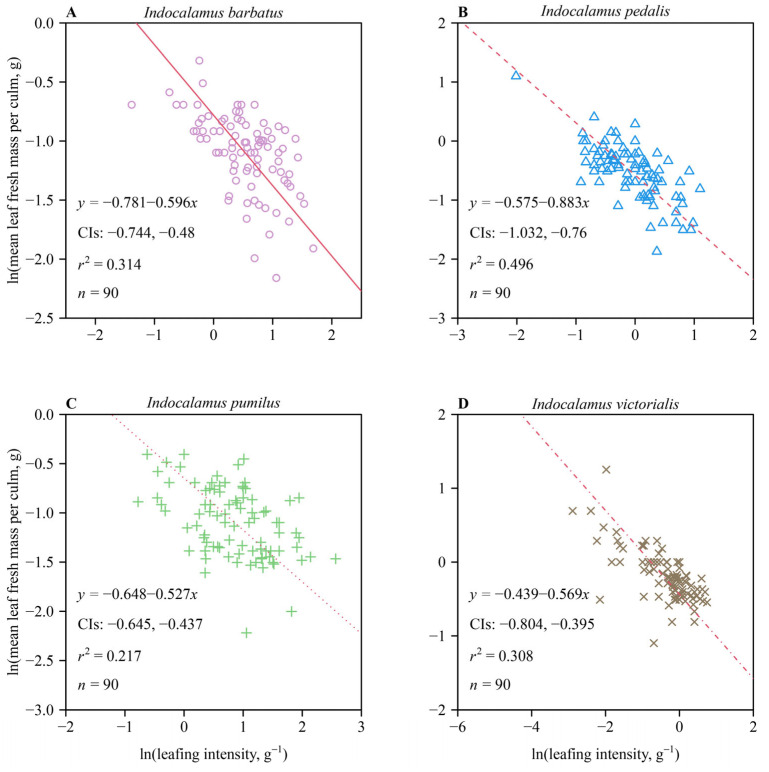
Fitted results for bivariate plots of mean leaf fresh mass per culm vs. leafing intensity on a log–log scale for four species of *Indocalamus*: *I. barbatus* (**A**), *I. pedalis* (**B**), *I. pumilus* (**C**), and *I. victorialis* (**D**). The red lines are the regression curves; CIs represent the 95% confidence intervals of the slope; *r*^2^ is the coefficient of determination; and *n* is the number of culms sampled for each of the four species.

For each of the four species, the scaling exponent for MLM vs. leafing intensity was negative, indicating that MLM decreases with increasing leafing intensity. With the exception of *I. pedalis*, the lower bounds of the 95% CIs of the scaling exponents for MLM vs. leafing intensity were greater than negative unity (Figure 2A,C,D), indicating that the decreases in MLM kept pace with increases in leafing intensity for these species. In contrast, the 95% CIs of the scaling exponent of MLM vs. the leafing intensity of *I. pedalis* included negative unity (Figure 2B), indicating that decreases in MLM did keep pace with increases in leafing intensity (i.e., a one-to-one relationship was observed).

**Figure 3:**
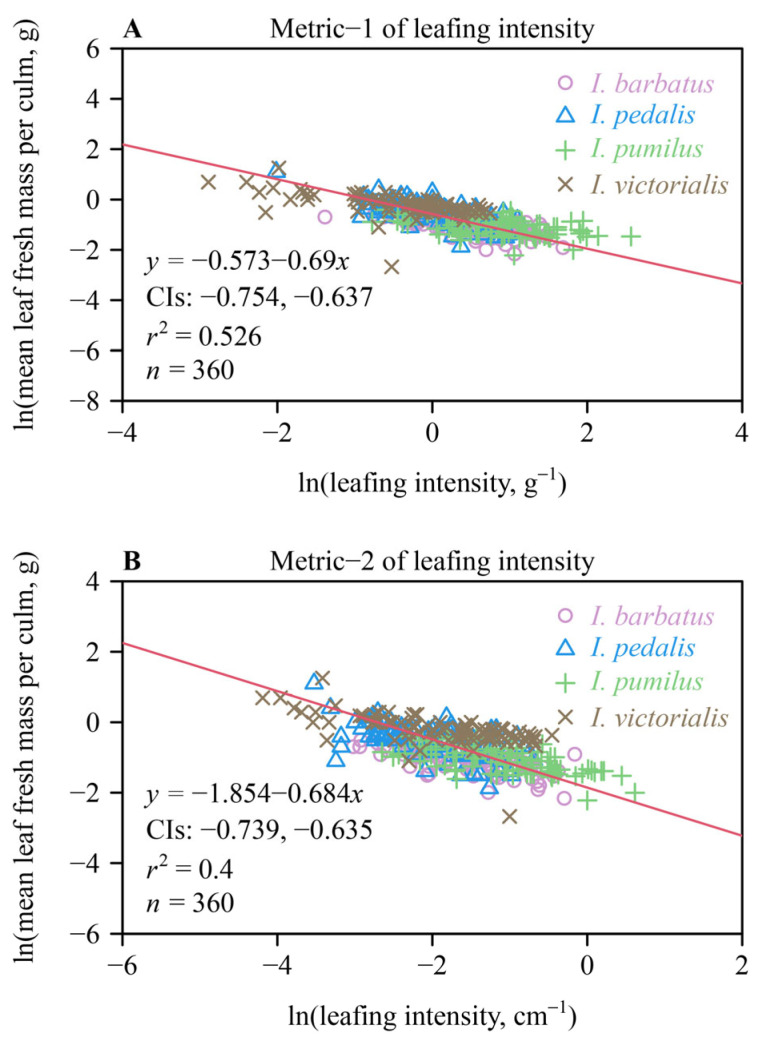
Fitted bivariate log–log scaling relationships for the pooled data of the four species of *Indocalamus*. (**A**) Mean leaf fresh mass per culm vs. leafing density from definition 1, and (**B**) the mean leaf fresh mass per culm vs. leafing density from definition 2. The red lines are the regression curves; CIs represent the 95% confidence intervals of the slope; *r*^2^ is the coefficient of determination; and *n* is the number of culms sampled for each species.

For both leafing intensity metrics (i.e., *N*/TNLM and *N*/*H*), the lower bounds of the 95% CIs of the scaling exponents for MLM vs. leafing intensity for the pooled data were greater than negative unity (Figure 3), indicating that decreases in MLM did not keep pace with increases in leafing intensity for all four species. The 95% CIs (i.e., −0.053 to 0.039) of the numerical difference in the scaling exponents of MLM vs. *N*/TNLM leafing intensity and those of the exponent of MLM vs. *N*/*H* leafing intensity included zero, indicating that there was no significant difference between the two scaling exponents. Therefore, both metrics for leafing intensity yielded the same results.

## 4. Discussion

This study focused on four species of the bamboo genus *Indocalamus* to explore the scaling relationships among important above-ground functional traits and to specifically examine the “leafing intensity premium” hypothesis, as well as to compare its two different definitions. The following sections discuss the implications of the results in the context of existing scaling theories and the literature, highlighting the ecological and evolutionary significance of leaf size variation and biomass investment strategies in *Indocalamus* species.

### 4.1. Scaling Relationship between TLM and TNLM

Among the four *Indocalamus* species, three exhibit isometric (one-to-one) scaling relationships between TLM and TNLM, i.e., the 95% CIs of the scaling exponents of TLM vs. TNLM for these three species all include unity. This is consistent with previous studies which found that in the case of species lacking substantial quantities of secondary tissues, leaf mass scales isometrically with respect to stem mass [39,40]. However, in the case of *I. pumilus*, an allometric relationship exists between TLM and TNLM, i.e., the upper bound of the 95% CIs of the corresponding scaling exponent was smaller than unity. This result indicates that increasing leaf mass requires a disproportionately larger investment in culm mass, reflecting the phenomenon called “diminishing returns” [27,41]. Indeed, many, if not most, studies have confirmed the phenomenon of “diminishing returns” when using the metric of dry leaf mass [27,29]. Arguably, the dry mass metric highlights the importance of carbon allocation, whereas fresh mass highlights the importance of mechanical support, since lamina water mass contributes to the total load a petiole must support [42,43]. Indeed, prior studies have shown that the lamina fresh mass vs. leaf surface area scaling relationship is typically statistically more robust than that of the lamina dry mass vs. leaf surface area scaling relationship, indicating that lamina fresh mass is a more biologically realistic indicator of the physiological processes and mechanics of leaves [44,45,46,47].

Clearly, leaves acquire carbon through photosynthesis, while stems provide mechanical support and transport water and nutrients to the leaves. Thus, a high degree of biomechanical and physiological coordination is anticipated between leaf and stem traits [21,48]. However, stems not only bear the static weight of the leaves but also dynamic forces such as wind [49]. The fact that bamboo culms are in fact stems helps to explain a “diminishing returns” phenomenon between TLM and TNLM.

### 4.2. Scaling Relationships between MLM and Leafing Intensity

For each of the four species, the scaling exponent of MLM vs. leafing intensity was negative, indicating that MLM decreases with increasing leafing intensity. A negative isometric relationship for MLM vs. leafing intensity was observed in the case of *I. pedalis*. This result is consistent with previous research indicating that an isometric trade-off exists between leaf size and leafing intensity associated with a constant biomass partitioning between leaves and stems [20]. The other three species and the pooled data for the four species exhibit negative allometric relationships between MLM and leafing intensity, indicating that the rate of increasing leafing intensity exceeds the rate of decreasing MLM. It is worth noting that leafing intensity can reflect the size of a plant’s “bud bank” [50]. As noted, species often require and use larger “bud banks” to compensate for their short stature and therefore manifest higher leafing intensities [33]. Leafing intensity may also provide a mechanism for “space escape” and “temporal escape” [33]. Plants with higher leafing intensities (and thus relatively more small leaves) can maximize the likelihood that at least some leaves will go unnoticed by insects, thus providing modest protection against herbivory [8,51]. As a “temporal escape” mechanism, plants that produce more leaves can gradually display their leaves over longer periods of time, allowing them to compensate for leaf tissue losses that occur early in the growing season [8,51,52].

### 4.3. Different Metrics of Leafing Intensity

Leafing intensity is commonly defined as the number of leaves per unit stem volume or mass [14,20,33]. However, stem volume and mass are closely correlated with plant height, which can be measured more conveniently and non-destructively compared to stem volume and mass. In this study, no difference was observed between the numerical values of the scaling exponents of the MLM vs. *N*/TNLM scaling relationship and the MLM vs. *N*/*H* scaling relationship (i.e., the 95% CI of the differences between the two groups of bootstrapping scaling exponent replicates included zero). This result indicates that *N*/*H* can be effectively used as a measure of leafing intensity for *Indocalamus* species. Future work is required to determine if this holds true for other monocot species or possibly eudicot species with more complicated leaf shapes.

## 5. Conclusions

This study investigated the scaling relationships among various above-ground functional traits associated with the “leafing intensity premium” hypothesis using four *Indocalamus* species. The results reveal both isometric and allometric scaling relationships between total leaf fresh mass per culm (TLM) and total non-leaf above-ground fresh mass (TNLM). These findings highlight complex resource allocation patterns for these bamboo species, with three of the four species exhibiting a phenomenon called “diminishing returns” in leaf mass investment. Additionally, negative scaling relationships between mean leaf fresh mass per culm (MLM) and leafing intensity were observed, indicating trade-offs between leaf size and number. Although *I. pedalis* displayed a negative isometric relationship, indicating a one-to-one biomass partitioning between MLM and leafing intensity, the other three species and the pooled data manifest negative allometric relationships, indicating a faster increase in leafing intensity compared to leaf size reduction. Finally, an alternative and equally effective definition of leafing intensity is validated (i.e., *N*/*H*), permitting a non-destructive assessment of leafing intensity for the four *Indocalamus* species. This work offers additional insights into the ecological and evolutionary significance of leaf size variation and biomass investment strategies in four *Indocalamus* species. Future research is needed to test whether the leafing intensity premium hypothesis holds true for other monocot and eudicot species, and whether there are significant differences in the scaling exponent of mean leaf size per shoot vs. leafing intensity for the same species growing under different conditions.

## Figures and Tables

**Table 1:** Morphological and agronomic characteristics of the four bamboo species.

Latin Name	Leaf Length (cm)	Total Number of Leaves	Total Leaf Fresh Mass (g)	Culm Fresh Mass (g)	Culm Height (cm)
*Indocalamus barbatus*	14.7 ± 4.2	21.4 ± 13.6	6.83 ± 4.00	18.7 ± 8.7	88.8 ± 26.6
*Indocalamus pedalis*	18.4 ± 5.6	11.0 ± 6.2	6.53 ± 3.90	18.1 ± 9.80	71.6 ± 23.2
*Indocalamus pumilus*	14.0 ± 4.1	23.4 ± 13.2	7.80 ± 4.42	19.7 ± 13.2	68.1 ± 35.4
*Indocalamus victorialis*	17.0 ± 4.3	14.2 ± 9.8	10.6 ± 6.42	29.2 ± 14.5	76.5 ± 23.5

Here, leaf length was estimated for each species without differentiating among individual plants of the same species, while other characteristics were measured for individual plants.

## Data Availability

The data can be found in the online Supplementary Table S1.

## Supplementary Materials

The following supporting information can be downloaded at: https://www.mdpi.com/article/10.3390/plants13162340/s1, Table S1: The raw data on various above-ground functional traits of four species of the genus *Indocalamus* Nakai.
